# Evaluating the Association Between Comorbidities and COVID-19 Severity Scoring on Chest CT Examinations Between the Two Waves of COVID-19: An Imaging Study Using Artificial Intelligence

**DOI:** 10.7759/cureus.21656

**Published:** 2022-01-27

**Authors:** Pranav Ajmera, Amit Kharat, Satvik Dhirawani, Sanjay M Khaladkar, Viraj Kulkarni, Vinay Duddalwar, Purnachandra Lamghare, Snehal Rathi

**Affiliations:** 1 Radiology, Dr. DY Patil Medical College, Hospital and Research Centre, Pune, IND; 2 Musculoskeletal Radiology, Dr. DY Patil Medical College, Hospital and Research Centre, Pune, IND; 3 Radiodiagnosis, Dr. DY Patil Medical College, Hospital and Research Centre, Pune, IND; 4 Data Science, DeepTek Medical Imaging Pvt. Ltd., Pune, IND; 5 Radiology and Biomedical Imaging, Keck School of Medicine, University of Southern California, Los Angeles, USA; 6 Radiology, Mahatma Gandhi Mission Institute of Health Sciences, Mumbai, IND

**Keywords:** radiology, artificial intelligence, ct chest, imaging, covid-19

## Abstract

Background

Coronavirus disease 2019 (COVID-19) has accounted for over 352 million cases and five million deaths globally. Although it affects populations across all nations, developing or transitional, of all genders and ages, the extent of the specific involvement is not very well known. This study aimed to analyze and determine how different were the first and second waves of the COVID-19 pandemic by assessing computed tomography severity scores (CT-SS).

Methodology

This was a retrospective, cross-sectional, observational study performed at a tertiary care Institution. We included 301 patients who underwent CT of the chest between June and October 2020 and 1,001 patients who underwent CT of the chest between February and April 2021. All included patients were symptomatic and were confirmed to be COVID-19 positive. We compared the CT-SS between the two datasets. In addition, we analyzed the distribution of CT-SS concerning age, comorbidities, and gender, as well as their differences between the two waves of COVID-19. Analysis was performed using the SPSS version 22 (IBM Corp., Armonk, NY, USA). The artificial intelligence platform U-net architecture with Xception encoder was used in the analysis.

Results

The study data revealed that while the mean CT-SS did not differ statistically between the two waves of COVID-19, the age group most affected in the second wave was almost a decade younger. While overall the disease had a predilection toward affecting males, our findings showed that females were more afflicted in the second wave of COVID-19 compared to the first wave. In particular, the disease had an increased severity in cases with comorbidities such as hypertension, diabetes mellitus, bronchial asthma, and tuberculosis.

Conclusions

This assessment demonstrated no significant difference in radiological severity score between the two waves of COVID-19. The secondary objective revealed that the two waves showed demographical differences. Hence, we iterate that no demographical subset of the population should be considered low risk as the disease manifestation was heterogeneous.

## Introduction

In December 2019, a few cases presented with pneumonia of unidentified origin at hospitals in Wuhan, China [1]. The apex case in India was detected on January 27, 2020, and the disease was declared as a Public Health Emergency on January 30, 2020 [2]. By February 11, 2020, the World Health Organization (WHO) had officially designated the disease as coronavirus disease 2019 (COVID-19). Meanwhile, COVID-19 had spread rapidly across many nations throughout the globe. The International Committee on Taxonomy of Viruses labeled this new type of coronavirus as severe acute respiratory syndrome coronavirus 2 (SARS-CoV-2), which is a type of beta coronavirus, an RNA virus [3,4].

While the spread has been unexpectedly rapid, the world has in the past decade dealt with a few other similar severe outbreaks such as the Ebola outbreak and the Middle East respiratory syndrome. Building upon the response chain from these diseases, the WHO, in sync with country-wise specific apex governmental organizations and institutions, activated its research and development blueprint to accelerate research, both diagnostic and therapeutic, to develop means for combating the pandemic [5].

As of January 24, 2022, the total number of cases globally stood at a staggering 352 million, with 279 million having recovered and nearly 5.6 million having succumbed to the disease. The daily average of cases and deaths globally from January 2020 to April 2021 peaked during the autumn of 2020 with another peak starting since February 2021, which have been termed as the first COVID-19 and the second COVID-19 waves, respectively [6]. Toward the end of 2020, it became apparent that there were going to be subsequent waves of COVID-19 with peaks distributed temporally in different countries. The largest contributory factor was human behavior in the form of a lack of regard toward restricted gatherings. Moreover, it was also observed that every time a country initiated de-restriction measures, after a lag of a couple of weeks from these measures, there was another COVID-19 wave with an exponential increase in the number of diagnosed cases [7]. While initially the disease presented with symptoms of fever and breathlessness, over weeks it was observed to cause symptoms in various organs with reports of gastric symptoms, bleeding manifestations, cardiac involvement, and septic shock among a few [8,9].

Borne out of extensive research for a diagnostic test was the reverse transcription-polymerase chain reaction (RT-PCR) and antigen-based rapid antigen test (RAT). While RAT has a high positive predictive value, it has a low negative predictive value, thus requiring another test to confirm. RT-PCR continues to be the gold standard test for COVID-19 [10]. However, while the test carries high specificity, it does not have enough sensitivity. With the increase in patient load, in developing economies, laboratories were under severe strain, leading to a significant backlog and shortage of kits, which led to a subsequent significant increase in the turnaround time of RT-PCR results. This acute gap was bridged by chest radiographs and chest computed tomography (CT) [10,11]. CT of the chest is easy to perform, scan time is a few minutes, and the subsequent evaluation is rapid. The use of disease-specific scores reduces interobserver variability and achieves high sensitivity in patients with moderate-to-severe symptoms. There have been enough studies to establish the typical signs on CT as bilateral ground-glass opacities (peripheral distribution > central; lower lobar > upper lobar involvement), crazy-paving, and consolidation [12]. Studies have shown that the sensitivity of CT is higher compared to RT-PCR, with a 98% sensitivity of CT compared to 71% of RT- PCR. Hence, there are high chances that an RT-PCR-negative patient can have CT features that may be typical of COVID-19 [11-13]. An umbrella review performed by Park et al. showed that the sensitivity of chest CT is particularly higher among symptomatic patients vis-a-vis where the majority of the patients were asymptomatic. In children, the sensitivity of chest CT is lower at around 70% [14].

Recent work has focused on artificial intelligence (AI), and its role in augmenting healthcare experts in the analysis of CT scans has been documented. AI can assist clinicians to triage the studies, localize disease, and even quantify and score lesions seen in COVID-19, thus making the reporting process objective and prioritizing reading for scans having higher scores [15,16]. In this study, we utilized AI to assess the demographic distribution and severity of COVID-19 infections through both the waves of the COVID-19 pandemic to provide valuable clinical insights which may be of use to policymakers to undertake preparations for the future onslaught of COVID-19 infections.

## Materials and methods

Patient cohort

**Figure 1:**
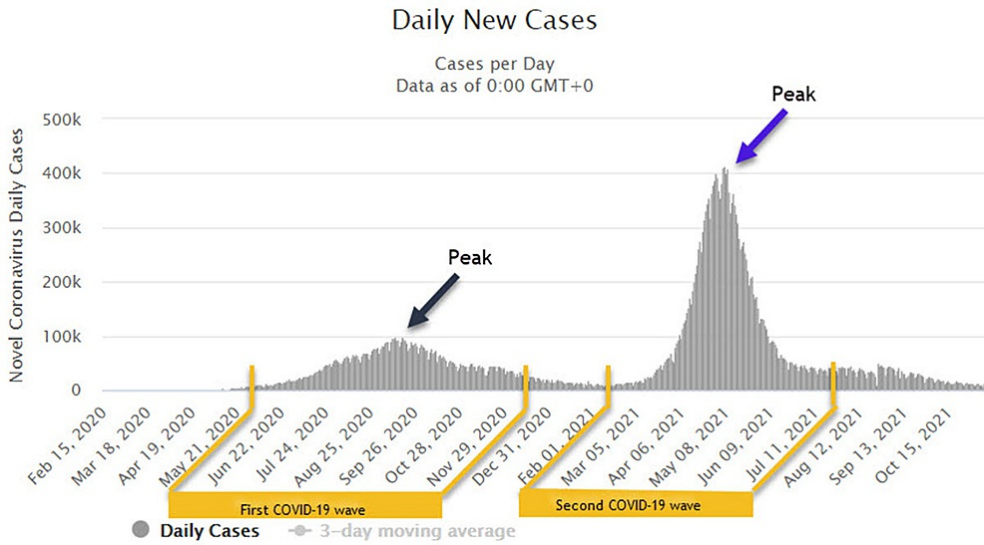
Average number of daily cases (above) between January 2020 and October 2021. The duration with the maximum caseload and peak of infection was selected for comparison. In the case of India, as the graph depicts, this period was between June and November 2020 for the first COVID-19 wave and March and July 2021 for the second COVID-19 wave. Data from worldometer.com was accessed on November 11, 2021. COVID-19: coronavirus disease 2019

The Institutional Review Board of Dr. DY Patil Medical College, Hospital and Research Centre, Dr. DY Patil Vidyapeeth University (our tertiary care hospital and referral center in Pune, Maharashtra, Western India) approved this retrospective study as part of the ethics subcommittee meet (DYPV/EC/596/2020). The data were anonymized and explicit written informed consent was waived in view of the retrospective nature of the data collection and usage of completely anonymized patient datasets. Subsequently, the periods when the first and second COVID-19 waves in India peaked were identified by data from www.worldometers.info/coronavirus/country/india/. For India, the period that saw the peak number of infections and was subsequently labeled as the first COVID-19 wave was June to November 2020. While the peak load of the second COVID-19 wave was seen in most states between March 2021 and July 2021 (Figure 1).

Data collection

**Figure 2:**
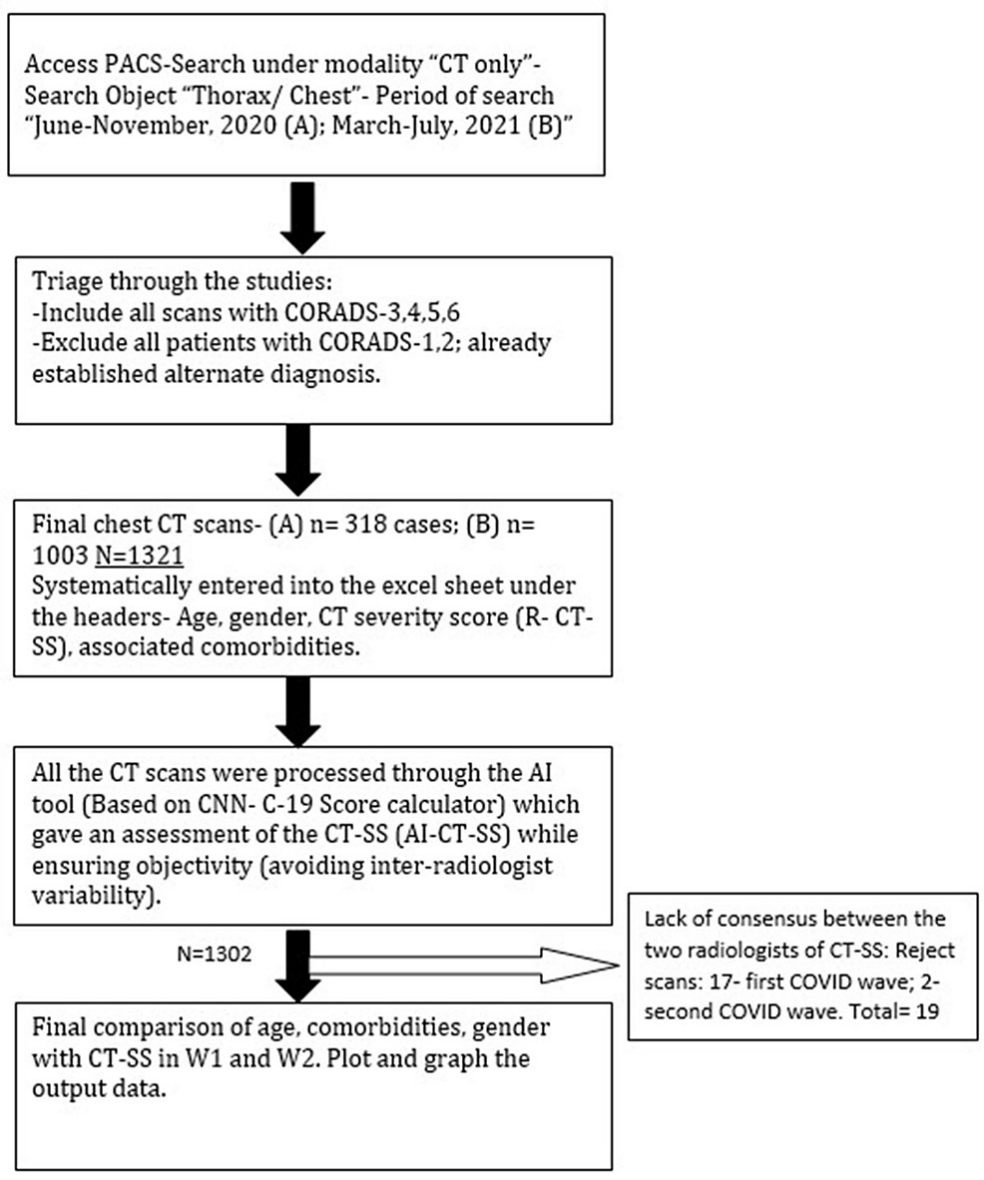
A flowchart depicting the stepwise research methodology utilized for data extraction, assessment, and interpretation. PACS: Picture Archiving and Communication System; CT: computed tomography; CORADS: COVID-19 Reporting and Data System; COVID-19: coronavirus disease 2019

All patients who underwent non-contrast chest CT during the periods June-November 2020 and March-July 2021 were identified on the Picture Archiving and Communication System (PACS) of the hospital utilizing the search feature. The CT scans were acquired at our institute on the SOMATOM CT-16 slice scanner (Siemens, Munich, Germany) with the following scan protocol: 120 kVP with a tube current of 110 mAs, a pitch of 1.3 mm, and scan time of 23.5 seconds. Subsequently, two radiologists with seven and five years of experience were tasked with identifying all the studies using text mining features on the PACS that were reported using the COVID-19 Reporting and Data System (CORADS). CORADS 1 and 2 were excluded as these are non-COVID-19 with reference to the CORADS criteria (Table 1). The CORADS-3 and above studies were taken into consideration in the study cohort selection criteria. We have depicted the study methodology in Figure 2.

**Table 1:** The CORADS classification system developed by the Dutch Radiological Society. Prokop M, van Everdingen W, van Rees Vellinga T, et al.: CO-RADS: a categorical CT assessment scheme for patients suspected of having COVID-19-definition and evaluation. Radiology. 2020, 296:E97-E104. 10.1148/radiol.2020201473 [19]. This article is available via the PMC Open Access Subset for unrestricted re-use and analyses in any form or by any means with acknowledgment of the original source. CT: computed tomography; COVID-19: coronavirus disease 2019; CORADS: COVID-19 Reporting and Data System; PCR: polymerase chain reaction

CORADS	Level of suspicion	CT findings
CORADS-1	No	Normal or non-infectious abnormalities
CORADS-2	Low	Abnormalities consistent with non-COVID-19 infections
CORADS-3	Indeterminate	Could be COVID-19 infection but could be other infection also
CORADS-4	High	Findings suspicious for COVID-19
CORADS-5	Very high	Findings very typical of COVID-19
CORADS-6	PCR positive	COVID-19 confirmed

CT chest assessment

As a measure to reduce the risk of transmission of COVID-19, all CT chests were performed on the 16-slice Siemens SOMATOM scanner, which was the designated COVID-19 scanner using the hospital-designed protocol. Therefore, the study protocol was homogeneous throughout the dataset.

Both radiologists reviewed thin slices of the CT images in the lung window and actively looked and documented the findings which were suspicious for COVID-19, including ground-glass opacities, consolidation in a predominantly peripheral distribution, and preferential involvement of lower lobes. They entered the findings in a separate excel sheet. The scans they read were non-overlapping, and their role was limited to ensuring that all the scans included in datasets A and B were CORADS-3 and above. For CT severity scores (CT-SS), we utilized an AI tool [17,18]. This tool was been created and tested before undertaking this project. The deep learning-based AI tool ensured that there was objectivity in the assessment and quantification of the scores across the entire dataset of 1,321 patients, thus avoiding the impact of inter-radiologist scoring variability. Once AI-CT-SS had been performed for the entire datasets, the AI-CT-SS was reviewed by radiologists to check for any obvious discrepancies in the model’s assessment of CT-SS (expert in the loop model). In such cases, a consensus of the two radiologists (different from the previous ones) was accepted as the final outcome. If there was a lack of consensus for a scan, those were removed from the study. There were 19 such scans in this category (17 from the first wave data and two from the second wave). Therefore, the overall number of patient scans evaluated in this study were 301 from the first COVID-19 wave and 1,001 from the second COVID-19 wave. We have plotted the final graphs based on the AI-CT-SS, hereafter, referred to as simply CT-SS.

Artificial intelligence tool

**Figure 3:**
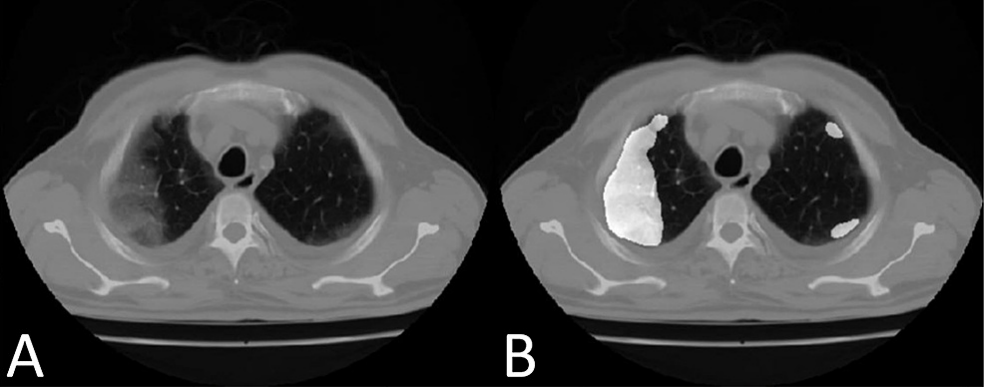
The deep learning tool used in detecting and scoring COVID-19 includes identifying the patch of the lung showing findings typical of COVID-19 (A) and then labeling it with a mask (B). The final output of the artificial intelligence tool is a CT severity score based on the sequential assessment of multiple CT slices. COVID-19: coronavirus disease 2019; CT: computed tomography

The AI tool we used to assign an objective CT-SS, as per the standard CT-SS system, was the deep learning-based quantification (scoring) algorithm (DeepTek Inc., Pune, India) (Figure 3).

Lokwani et al. have published the details of model development and training [17]. This is a two-dimensional (2D) model built on slice level. It is based on a U-net architecture of convolutional neural networks (CNN) for the segmentation of medical images; the encoder utilized for this purpose was the Xception encoder. The model was trained on the standard Adam Adaptive optimizer with binary cross-entropy as the loss function. Each image was resized to 512 × 512 pixels. The final output was in the form of CT-SS for each patient. The model helped in slice-level localization, while the U-Net with Attention Model was used for quantification of the scores. The tool was developed with the logic that 15 slices with a positive prediction of COVID-19 should be categorized as COVID-19 at the scan level. The number 15 was decided by the developers based on testing the model with different numbers during the training and validation of the model. Therefore, the integrated model could assess a CT scan and provide a scan-level prediction alongside a quantified CT-SS. This deep learning-based AI model had a sensitivity of 0.964, specificity of 0.884, and f-1 score of 0.794 on the test set.

Statistical analysis

Data were analyzed using SPSS version 22 software (IBM Corp., Armonk, NY, USA). Categorical data were represented as frequencies and proportions. The chi-square test was used as a test of significance for qualitative data. We represented continuous data as mean and standard deviation. Independent t-test or Mann-Whitney U-test was used as a test of significance to identify the mean differences between two quantitative variables and qualitative variables, respectively.

## Results

Patient characteristics

**Figure 4:**
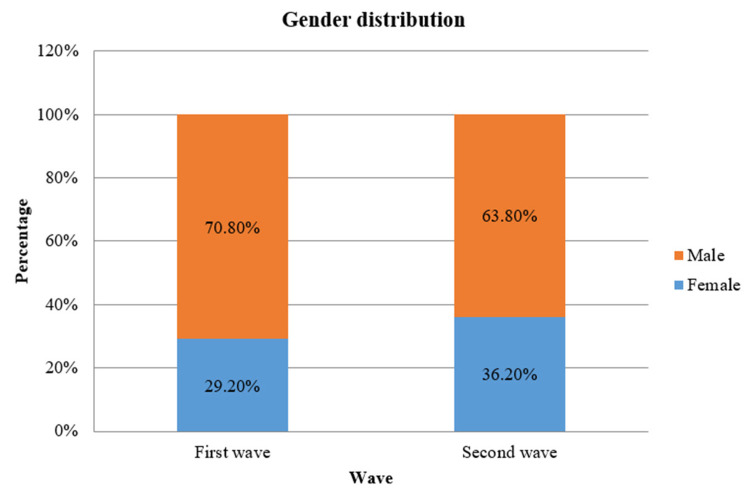
A bar diagram depicting the distribution of genders between the two waves of COVID-19. COVID-19: coronavirus disease 2019

Of the 301 patients in the first COVID-19 wave, 70.8% were males and 29.2% were females; in the second COVID-19 wave, the predilection for affecting females was higher at 36.2% and males at 63.8% (Figure 4). This difference in the involvement of more females in the second COVID-19 wave was statistically significant.

**Figure 5:**
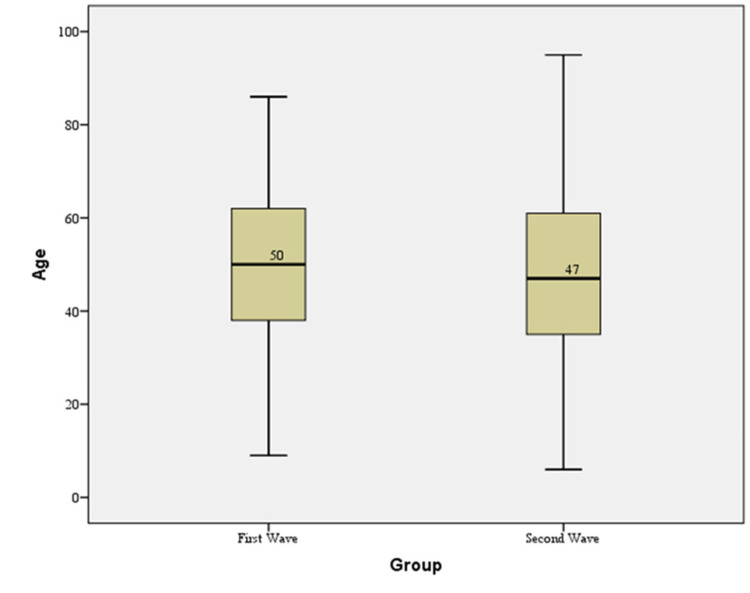
A box and Whisker plot comparison of the distribution of the mean age between the two waves of COVID-19. COVID-19: coronavirus disease 2019

The mean age of patients in the first COVID-19 wave was 50.32 ± 15.44 years and in the second COVID-19 wave was 48.08 ± 16.47 years, with a statistically significant difference in the mean age between the two COVID-19 waves (Figure 5, Table 2). In the first COVID-19 wave, the majority of patients were in the age group of 41 to 50 years (21.6%), and in the second COVID-19 wave, the majority of patients were in the age group of 31 to 40 years (22%).

**Table 2:** Comparison of the mean age between the two waves of COVID-19. COVID-19: coronavirus disease 2019; SD: standard deviation

		Group					
		First wave of COVID-19		Second wave of COVID-19		Total	
		Count	%	Count	%	Count	%
Age	<10 years	2	0.7%	3	0.3%	5	0.4%
	11–20 years	1	0.3%	14	1.4%	15	1.2%
	21–30 years	28	9.3%	146	14.6%	174	13.4%
	31–40 years	60	19.9%	220	22.0%	280	21.5%
	41–50 years	65	21.6%	193	19.3%	258	19.8%
	51–60 years	59	19.6%	169	16.9%	228	17.5%
	61–70 years	56	18.6%	167	16.7%	223	17.1%
	71–80 years	25	8.3%	67	6.7%	92	7.1%
	81–90 years	5	1.7%	20	2.0%	25	1.9%
	>90 years	0	0.0%	2	0.2%	2	0.2%
	Mean ± SD	50.32 ± 15.441		48.08 ± 16.479		P = 0.036*	

In the dataset reviewed, 2% had bronchial asthma, 17.3% had diabetes mellitus, 24.3% had hypertension, and 1% had active tuberculosis in the first COVID-19 wave. The second COVID-19 wave had a similar distribution of cases with comorbidities, 1.5% had bronchial asthma, 15% had diabetes mellitus, 20.5% had hypertension, 0.9% had active tuberculosis. Hence, there was no significant difference in the distribution of comorbidities between the two COVID-19 waves.

As per the CORADS distribution, only 10.7% of all cases in the first COVID-19 wave and 11.2% of all cases in the second COVID-19 wave belonged to the CORADS-3 and 4 categories combined. Overall, 33.2% of cases in the first COVID-19 wave and 22.1% of cases in the second COVID-19 wave were categorized as CORADS-5, while 56.1% and 66.7% cases in the first and second COVID-19 waves, respectively, were RT-PCR-confirmed COVID-19 cases (CORADS-6) (Table 3).

**Table 3:** Comparison between CT severity score and comorbidities in the first and second waves of COVID-19. Chi-sqaure = 18.69, df = 3; p < 0.001* (chi-square test). CORADS: COVID-19 Reporting and Data System; CT: computed tomography; COVID-19: coronavirus disease 2019

		Group					
		First wave		Second wave		Total	
		Count	%	Count	%	Count	%
CORADS	3	11	3.7%	58	5.8%	69	5.3%
	4	21	7.0%	54	5.4%	75	5.8%
	5	100	33.2%	221	22.1%	321	24.7%
	6	169	56.1%	668	66.7%	837	64.3%

CT severity scores

**Figure 6:**
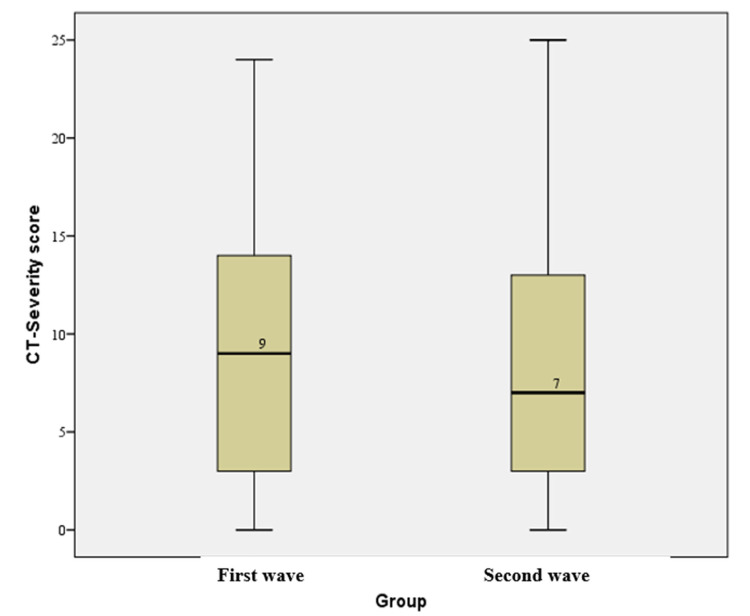
A box and whisker plot comparison of CT severity scores between the first and second waves of COVID-19. COVID-19: coronavirus disease 2019

The median CT-SS was 9 and 7 for the first and second COVID-19 waves, respectively; however, this difference was not statistically significant (p > 0.05) on assessment using the Mann-Whitney U-test (Figure 6). The mean CT-SS in the first COVID-19 wave was 9 and in the second COVID-19 wave was 8.3.

**Figure 7:**
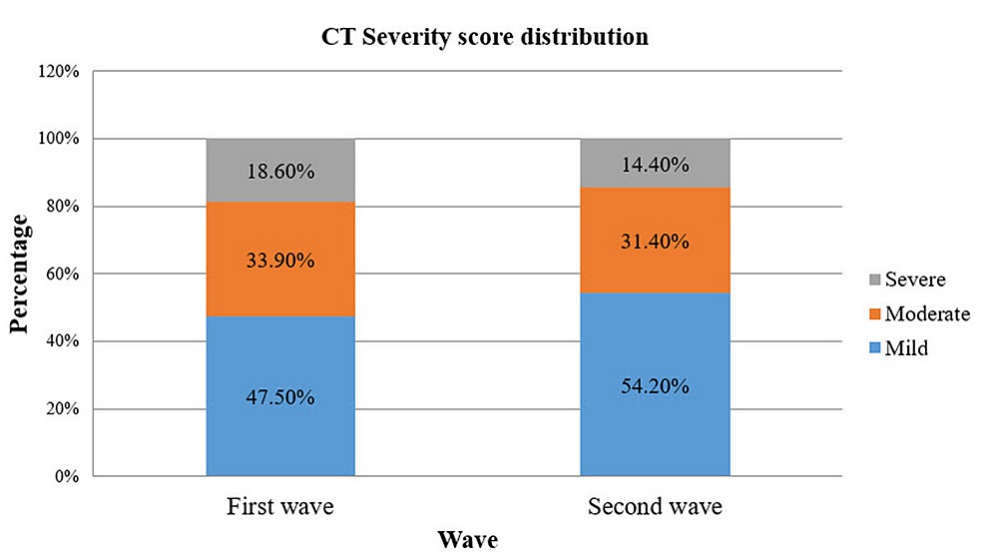
A bar diagram depicting the distribution of CT severity scores in the first and second waves of COVID-19. CT: computed tomography; COVID-19: coronavirus disease 2019

Based on mild, moderate, and severe criteria of CT-SS, the data revealed that while 47.5% of patients in the first COVID-19 wave were mild compared to 54.2% of patients in the second COVID-19 wave, 33.9% were in the moderate category in the first COVID-19 wave and 31.4% in the second COVID-19 wave. The figures for the severe category were 18.6% and 14.4% in the first and second COVID-19 waves, respectively (Figure 7).

Impact of comorbidities

The most commonly associated comorbidities in the study population were diabetes mellitus, hypertension, bronchial asthma, and tuberculosis, with isolated diabetes mellitus and hypertension having a statistically significant association (p < 0.05) with higher CT-SS. Comparisons between CT-SS and comorbidities between the two COVID-19 waves are presented in Table 4.

**Table 4:** CT severity score with respect to comorbidities in the first and second waves of COVID-19. CT: computed tomography; COVID-19: coronavirus disease 2019; SD: standard deviation

		CT severity score										
		First wave of COVID-19					Second wave of COVID-19					
		N	Mean	SD	Median	P-value	N	Mean	SD	Median	P-value	
Bronchial asthma	Yes	6	10.7	5.5	11	0.508	15	7.1	5.0	7	0.477	
	No	295	9.0	6.2	9		986	8.3	6.2	7		
Diabetes mellitus	Yes	52	10.8	6.1	12	0.022*	150	10.5	5.9	11	<0.001*	
	No	249	8.6	6.2	8		851	7.9	6.1	7		
Hypertension	Yes	73	11.3	5.9	12	<0.001*	205	10.2	5.8	10	<0.001*	
	No	228	8.3	6.1	8		796	7.7	6.1	7		
Tuberculosis	Yes	3	15.3	1.2	16	0.076	9	10.0	6.5	9	0.391	
	No	298	8.9	6.2	9		992	8.2	6.1	7		

In the first COVID-19 wave, among patients with diabetes mellitus, the median CT-SS value was 12, and among patients without diabetes mellitus, it was 8. Similarly, in the second COVID-19 wave, among patients with diabetes mellitus, the median CT-SS was 11, and among patients without diabetes mellitus, it was 7. In the first COVID-19 wave, among patients with hypertension, the median CT-SS was 12, and among patients without hypertension, it was 8. Similarly, in the second COVID-19 wave, among patients with hypertension, the median CT-SS was 10, and among patients without hypertension, it was 7. These values were statistically significant in both waves.

## Discussion

The early diagnostic tools for COVID-19 were based on clinical assessment along with an entire blood panel, especially inflammatory markers such as C-reactive protein, erythrocyte sedimentation rate, and D-dimer [19,20]. With rapid research, RT-PCR was developed against COVID-19. While it has high specificity, it continues to suffer from low sensitivity and needs supplementation with imaging. Imaging includes chest radiography and chest CT, but radiographs are insensitive to mild and even moderate disease or those categories of patients who do not have many respiratory symptoms; hence, radiographs are less sensitive vis-à-vis CT scans [21-23].

To objectively assess the risk of a patient being afflicted by the disease, the CORADS was developed by the Dutch Radiological Society. This standardized reporting system was developed and functionalized in early 2020 after a recommendation by the Fleischner Society which issued as part of the consensus statement on COVID-19 that imaging has a significant role to play in the workup. Previous experiences with other standardized reporting formats such as the Lung Imaging and Reporting System and the Breast Reporting and Imaging System have made it clear that structured reporting helps in objectifying reports and ensures a basic standard of scan assessment. The CORADS system has categories ranging from 0 to 6 [19,24].

Findings typical of COVID-19 on a thoracic CT encompass ground-glass opacities (multifocal > single; peripheral > hilar), consolidation, linear opacities, and crazy paving pattern. Findings such as vascular enlargement, white-out lung, and air-bronchogram can be indicative of COVID-19. All other signs such as cavitation, pulmonary nodule(s), lymphadenopathy, the halo sign, the tree-in-bud sign, bronchiectasis, pulmonary emphysema, pulmonary fibrosis, isolated pleural thickening, pneumothorax, and pericardial effusion are atypical signs suggesting exploration for an alternate diagnosis [25-27].

Currently, the available literature on the assessment and analysis of the first and second COVID-19 waves of the pandemic is very limited. A PubMed search for previous papers on imaging comparison between the two waves was performed utilizing the search strings “COVID-19,” “Coronavirus-19,” “First and second COVID-19 waves,” and “CT severity scores” on November 18, 2021, at 9:30 AM IST. Although the search did not yield any relevant published papers focussed on the imaging perspective, we did obtain a few articles comparing the mortality and spread of the disease between the two COVID-19 waves in a few Western countries.

The data published by Seligmann et al. suggested that the second wave of COVID-19 was associated with a younger age group of the affected population [28]. These findings support the findings of our study which showed that the population most affected in the second COVID-19 wave was almost a decade younger than the previous COVID-19 wave.

In one of the papers published on the analysis of mortality data from the United States and Europe, the data revealed that while the caseload was more in the second COVID-19 wave, there was a reduction in mortality in the second wave [29]. Loannidis et al. assessed whether the age distribution of COVID-19 deaths and the share of deaths in nursing homes changed in the second versus the first COVID-19 waves. Their data revealed that there was not much difference in the distribution of deaths with differing age groups in both waves. Unfortunately, our study was limited in not having access to mortality data for patients [30].

In our study, the mean CT-SS in cases with comorbidities was higher, with diabetes mellitus and hypertension being the most common comorbidities associated with higher scores. These findings match the data from a meta-analysis performed by Zhou et al. on the association of these risk factors with more severe clinical outcomes [31].

While our patient cohort is large with 1,302 patients, a relative limitation is that it is a single-center study. The datasets we used for the first and second COVID-19 waves are imbalanced, that is, 301 for the first COVID-19 wave and 1,001 for the second COVID-19 wave, because in the first wave chest CT was not utilized on a large scale as a diagnostic tool; therefore, the datasets were limited. We did not include the eventual patient outcomes or assess their hospital stay, but these will subsequently be assessed. It is vital that more studies are performed that can, to varying extents, utilize the available clinical, morbidity, and mortality data because this is the key to gaining important clinical insights.

## Conclusions

In our study, the analysis of the COVID-19 waves has revealed that while the mean CT-SS was not significantly different between the two waves of COVID-19 in India, COVID-19 affected more individuals from a younger age group in the second COVID-19 wave. Moreover, the second COVID-19 wave was associated with an increased affliction of females. Overall, comorbidities had a significant impact on the CT-SS, with higher scores seen in the comorbidities group than in the population cohort without any comorbidities. Utilizing AI ensured a high degree of objectivity by avoiding the errors arising out of inter-radiologist variability on CT COVID-19 scoring. This assessment provides insights in comprehending both COVID-19 waves by evaluating the differences in the demographic composition of the population affected and the association between the presence of comorbidities and the severity of affliction using radiological COVID-19 CT scoring as the basis.
